# The effect of intestinal microbiota metabolites on HT29 cell line using MTT method in patients with colorectal cancer 

**Published:** 2019

**Authors:** Somayeh Jahani-Sherafat, Masoumeh Azimirad, Hajieh Ghasemian-Safaei, Hadi Ahmadi Amoli, Sharareh Moghim, Ghazal Sherkat, Mohammad Reza Zali

**Affiliations:** 1 *Foodborne and Waterborne Diseases Research Center, Research Institute for Gastroenterology and Liver Diseases, Shahid Beheshti University of Medical Sciences, Tehran, Iran*; 2 *Department of Microbiology, School of Medicine, Isfahan University of Medical Sciences, Isfahan, Iran*; 3 *Sina Hospital, Tehran University of Medical Sciences, Tehran, Iran*; 4 *Student Research Committee, Islamic Azad University, Mashhad Branch, Mashhad, Iran*; 5 *Gastroenterology and Liver Diseases Research Center, Research Institute for Gastroenterology and Liver Diseases, Shahid Beheshti University of Medical Sciences, Tehran, Iran *

**Keywords:** Metabolites, Cell line, MTT, Colorectal cancer

## Abstract

**Aim:**

The aim of this study was to evaluate the effect of intestinal microbiota metabolites in colorectal cancer patients on HT29 cell line using MTT assay.

**Background:**

Colorectal cancer is one of the most common malignant tumors. Human guts harbor abundant microbes that adjust many aspects of the host physiology. Increasing studies suggest that gut microbiota play a significant role in the incidence and expansion of CRC, as a result of virulence factors, bacterial metabolites, or inflammatory pathways.

**Methods:**

In this cross-sectional study, 60 biopsy samples including 30 cancerous and 30 adjacent healthy tissues were collected from patients with CRC during 2017. Biopsy samples were first cultured on Thioglycollate broth medium for 24hr after which the microbiota metabolites were filtered and stored at -20 C° for further evaluation. HT29 cells were treated by microbiota metabolites at different times (3, 6, 12, 18h) and its viability was assessed by MTT assay.

**Results:**

The cells treated with microbiota metabolites showed increased viability and proliferation in time-dependent analysis by MTT assay, but there was not significant differences between the two groups.

**Conclusion:**

It seems that microbial metabolites are able to induce proliferation and increase cell viability and thus induce colorectal cancer.

## Introduction

 Colorectal cancer (CRC) is the third most common cancer and the fourth leading cause of cancer death in the world (1). CRC is the fourth most common cancer in men following gastric, bladder, and prostate cancers and is the second most common cancer in women after breast cancer in Iran (2). The incidence of CRC in Iran is 7-8 people per 100,000 (3) and in young people or early CRC it has been reported as 20%. This might be due to a change in lifestyle and an increase in meat and fat consumption and a reduction in the consumption of grains and fiber in the Iranian diet (3). Since the cause of colorectal cancer is mostly related to environmental and epigenetic factors, it is important to study the effect of environmental factors on CRC progression.

Among environmental factors, microbiota have recently been given a very prominent role that can directly and indirectly facilitate the pathway of cancer by their structural components as well as their enzymes and metabolites as well as the production of oxygen free radicals (4). Microbial imbalance in the gut (dysbiosis) can play a key role in altering the composition of intestinal bacterial components. High production of some harmful bacterial enzymes, alteration in the distribution of bacterial communities (commensal bacteria), changes in bacterial metabolic activity (SCFA), the metabolism of bile acids, and loss of protection against dietary carcinogens are the result of dysbiosis (5). (6). Many factors, including antibiotic usage, psychological and physical stress, radiation, altered bowel motility and diet can alter the digestive ecosystem and thus change the bacterial composition (7, 8). On the other hand, some intestinal anaerobic bacteria, such as *Clostridium* and *bacterioides*, as the predominant population of the intestinal microbiota, produce bacterial metabolites such as bile acids, fatty acids, β-galactosidase, β-glucosidase, reductase, decarboxylase, and protease which can also increase the risk of colorectal cancer (9). It has been reported that about 30% of *Clostridium* were capable of producing high levels of β-galactosidase enzymes (10). The level of this enzyme has been reported to be far higher in the stools of patients with colorectal cancer as compared with healthy controls (11). Azoreductase can be involved in the development of cancer by producing toxic substances from dyes and drugs. This enzyme is active in bacteria such as *Bacteroides fragillis* and *Beta thioatomicron* and some *Clostridium* such as *Clostridium perfringens* (12). Some bacteria, such as *Bacteroides, Streptococcus*, and *Clostridium*, produce high levels of proteolytic enzymes, such as proteasomes, which can bind to intestinal epithelial cells by specific receptors and cause binding, biofilm formation, and invasion of these cells. This is a first step towards the disease, which is inflammation (13, 14). By considering previous studies on the role of bacteria and metabolites produced by them, it may be possible to find a link between CRC and metabolites produced by the gut microbiota. Multiple in vitro tests are trying to assess the toxicity of some compounds. The MTT (3-(4,5-dimethylthiazol-2-yl)-2,5-diphenyl tetrazolium bromide) is one of the most commonly used assays to assess the toxicity of some agents (15). This method is reliable for investigation of the influence of microbial metabolites on cytotoxicity or viability and cell proliferation (16). The aim of this study was to evaluate the effect of intestinal microbiota metabolites in colorectal cancer patients on HT29 cell line using MTT assay. 

## Methods

**Samples and preparation of microbiota metabolites:**

Thirty specimens of cancerous tissue and 30 specimens of adjacent healthy tissue were collected from patients with colon cancer during surgery. Tissue samples were transferred to the microbiology laboratory under anaerobic conditions in broth thioglycolate medium to study the metabolic effects of the bacteria on the cell culture medium. Samples were homogenized manually using a tissue grinder and then incubated under completely anaerobic conditions for 24 hours at 37 °C. It was then stored using a 0.22μm syringe filter and a bacterial-free material filter was stored at -20 °C for the effect of bacterial metabolites study.

**Cell culture**

To investigate the effect of bacterial metabolites on intestinal cells, the HT29 cell line originating from adenocarcinoma cells was used. This cell line was purchased from the Pasteur Institute of Iran. The culture medium used was H-DMEM containing 10% FBS (Sigma-Aldrich), 2% non-essential amino acid (NEAA, Gibco), and 1% antibiotics. The cells were passaged for several generations in special flasks using trypsin-EDTA (0.25%). Trypsinized cells were incubated at 37 ° C and 5% CO2 atmosphere.

**Figure 1:**
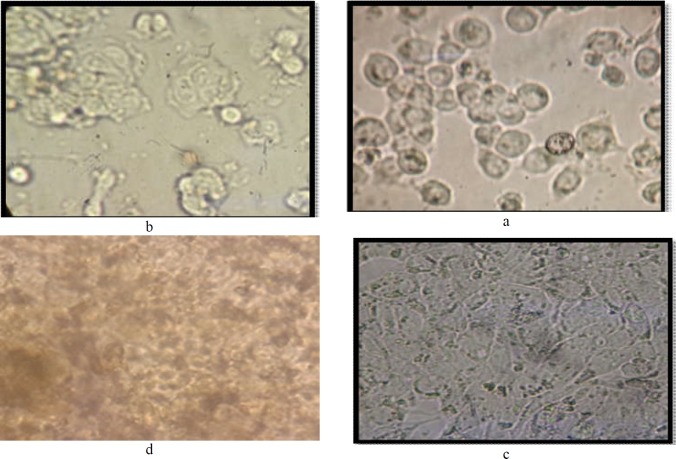
HT-29 cell line; a: before treatment with bacterial metabolites, a, b: Untreated cells during testing on 24-well plates, c: Differentiated cells ready for treatment by bacterial metabolites, d: Cells treated with bacterial metabolites for 24 hours

**Figure 2:**
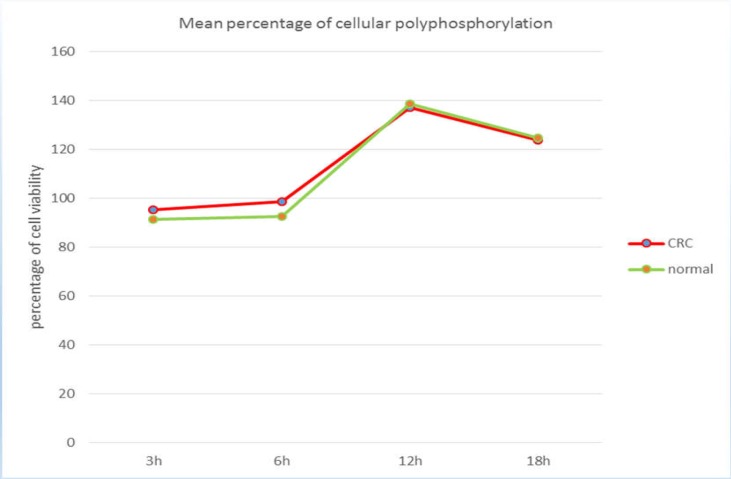
Comparison of the mean cell viability with microbiota metabolites at different time points in cancer and normal samples

**MTT test**

To investigate the effect of cytotoxicity or proliferation of metabolites obtained from microbiota of CRC patients, cell viability was assessed using MTT Assay kit (Sigma, America) after complete growth of HT29 cells for 3 days. A total of 100μ of filtered bacterial metabolites was shed on the cells and incubated at 3, 6, 12, and 18 hrs at 37° C as well as atmosphere containing 5% Co2. Thereafter, 100µL of MTT solution was added to the plates and incubated for 4 hours at 37°C and 5% Co2 atmosphere. Then, 1 ml of the solubilization solution was added to the plates and incubated 15-20 min at 37° C with a 5% Co2 atmosphere. Next, reads at 570 nm and 650 nm, as a reference wavelength, using ELISA Reader, (ELX808, Biotek). Cell viability was calculated using the following formula: Viability (%) = (OD test/OD control) x100

**Statistical analysis**

Statistical calculations were performed to compare the mean and standard deviation using SPSS Ver.20 and t-Test with P <0.05 considered statistically significant (P <0.05). 

## Results

The cultures of HT29 cells were treated with metabolites after cell growth and differentiation (Figure 1). MTT assay was performed in 3, 6, 12, and 18hr of simultaneous microbiota cell and metabolite culture (Figure 1).

The cytotoxic effect of bacterial metabolites on the HT29 cell line was assessed by ELISA reader on differentiated cell supernatants. These results confirm the lack of toxic effects of the investigated metabolites on differentiated cell lines at 6, 3, 12, 18 h (Figure 2). As depicted in Figure 5, the lowest percentage of cells exposed to metabolites and the highest proliferation rate were obtained at 3 h and 12 h, respectively. There was no significant difference between the results of cancerous tissue and adjacent healthy tissue (p =0.1).

## Discussion

Cancer is one of the leading causes of death worldwide. According to WHO projections by 2030, more than 21 million new cancer cases and more than 13 million cancer deaths will occur globally. Meanwhile, colon cancer is also considered to be the third most common cancer in the world (1). 

Microbial pathogens are known to initiate 20% of tumorigenesis and a considerable number of malignancies are associated with dysbiosis (17). Intestinal microbiota changes in patients with colorectal cancer have been reported in many studies, but what is controversial is whether the cancer is a product of a change in the microbiota or whether cancer progression causes changes in the natural microbiome. There is strong evidence for both theories (17, 18). The gut microbiota can promote CRC through a variety of processes, including inducing a chronic inflammatory disease or immune response, altering stem cell dynamics, biosynthesis of toxic metabolites, genotoxins, influencing host metabolism, and preventing cancer by producing metabolites and enzymes (20, 21).

In the present study, the role of intestinal microbiota in CRCs was investigated on the lethality or cellular proliferation on HT29 cells. Based on the results, the microbiota metabolites of these patients induced cell proliferation in cell culture compared to blank cell culture. The mean percentage of cellular proliferation in metabolites obtained from tumor specimens was slightly higher than in normal tissue samples but no significant difference was observed between the two groups. Both normal and tumor specimens were taken from one patient; although some studies have reported differences between microbiota in cancer and normal tissue, most studies have indicated that the structure of the gut microbiota often forms before three years of age and has a stable structure throughout their lives (22).

Certain factors such as antibiotic use, specific diet, chemotherapy, etc. can disrupt the gut microbiota structure (23). In a study conducted by Sadeghi and colleagues in 2018 on chromogenic acid along with microbial metabolites from fruits and vegetables, it was found that these microbial metabolites have an antiproliferative effect on the caco2 cell line and induced apoptosis in cells after 24 hours (16). In this study, anticancer agents were used and their results were in line with our results. In another study, Shi et al. (2016) studied the effects of LPS (lipopolysaccharide) on inflammatory factors using MTT assay. They found that LPS incubation time had a significant relationship with cellular proliferation factors. superoxide dismutase (SOD) levels were not significantly different between the experimental and control groups, but the catalase enzyme was significantly reduced. Cell proliferation at concentrations of 0.1 and 1 μg/L and 6 h incubation decreased cell proliferation, and this result is probably because of toxic concentration of LPS to cells thus causing cell death (24). Studies have shown that LPS causes oxidative damage and has been widely used to create animal models of inflammation and bacterial infection (25).

In a study examining effect of fecal filtered fluid on caco2 cell culture using comet test, Venturi and colleagues observed that in normal samples they had no detrimental effect on DNA breakage, but in samples with intestinal problems such as intestinal polyps, DNA breakage was observed. (26). DNA breakdown can cause carcinogenic mutations in the cell and as these mutations accumulate, the cells move toward cancer. On the other hand, some bacterial metabolites such as *Enterococcus faecalis, Enterotoxin bacteroides Fragillis* or fadA in *Fusobacterium nucleatum* have the ability to break down DNA and thus induce intestinal cell pre-proliferation and increase in intestinal microbiota in microbiota studies (27).

Given the effect of bacterial metabolites isolated from CRC patients on increasing proliferation of the HT29 cell line, it seems that bacterial metabolites may play a central role in the relationship between the microbiota and associated colon cancer. Bacteria can alter the expression of genes involved in carcinogenesis during infection or their inflammation or directly by the production of intestinal microbiota metabolites. More studies are required to study the role of gut microbiota in CRC patients.

## Conflict of interests

The authors declare that they have no conflict of interest.
